# Long-term risk and predictors of cerebrovascular events following sepsis hospitalization: A systematic review and meta-analysis

**DOI:** 10.3389/fmed.2022.1065476

**Published:** 2022-11-25

**Authors:** Amanuel Godana Arero, Ali Vasheghani-Farahani, Bereket Molla Tigabu, Godana Arero, Beniyam Yimam Ayene, Danesh Soltani

**Affiliations:** ^1^Cardiac Primary Prevention Research Center, Cardiovascular Diseases Research Institute, Tehran University of Medical Sciences, Tehran, Iran; ^2^Universal Scientific Education and Research Network, Addis Ababa, Ethiopia; ^3^Department of Clinical Cardiac Electrophysiology, Tehran Heart Center, Tehran University of Medical Sciences, Tehran, Iran; ^4^Department of Pharmacy, Komar University of Science and Technology, Sulaymaniyah, Iraq; ^5^Department of Public Health, Adama Hospital Medical College, Adama, Ethiopia; ^6^School of Medicine, College of Health Sciences, Addis Ababa University, Addis Ababa, Ethiopia; ^7^Students’ Scientific Research Center, Tehran University of Medical Sciences, Tehran, Iran

**Keywords:** sepsis, stroke, cerebrovascular events, cardiovascular events, meta-analysis

## Abstract

**Background:**

Long-term risk and predictors of cerebrovascular events following sepsis hospitalization have not been clearly elucidated. We aim to determine the association between surviving sepsis hospitalization and cerebrovascular complications in adult sepsis survivors.

**Method:**

We searched MEDLINE, Embase, Scopus, Web of Sciences, Cochrane library, and Google scholar for studies published from the inception of each database until 31 August 2022.

**Results:**

Of 8,601 screened citations, 12 observational studies involving 829,506 participants were analyzed. Surviving sepsis hospitalization was associated with a significantly higher ischemic stroke [adjusted hazard ratio (aHR) 1.45 (95% CI, 1.23–1.71), *I*^2^ = 96], and hemorrhagic stroke [aHR 2.22 (95% CI, 1.11–4.42), *I*^2^ = 96] at maximum follow-up compared to non-sepsis hospital or population control. The increased risk was robust to several sensitivity analyses. Factors that were significantly associated with increased hazards of stroke were: advanced age, male gender, diabetes mellitus, hypertension, coronary artery disease, chronic heart failure, chronic kidney disease, chronic obstruction pulmonary disease, and new-onset atrial fibrillation. Only diabetes mellites [aHR 1.80 (95% CI, 1.12–2.91)], hypertension [aHR 2.2 (95% CI, 2.03–2.52)], coronary artery disease [HR 1.64 (95% CI, 1.49–1.80)], and new-onset atrial fibrillation [aHR 1.80 (95% CI, 1.42–2.28)], were associated with > 50% increase in hazards.

**Conclusion:**

Our findings showed a significant association between sepsis and a subsequent risk of cerebrovascular events. The risk of cerebrovascular events can be predicated by patient and sepsis-related baseline variables. New therapeutic strategies are needed for the high-risk patients.

## Introduction

According to the third International consensus definition for sepsis and septic shock (Sepsis-3), sepsis is defined as a life-threatening organ dysfunction caused by a dysregulated host response to infection (1). Sepsis is a major public health challenge and is the cause of substantial mortality, cost, and healthcare utilization (2, 3). Beyond the acute hospital death, it has been demonstrated that patients who have survived sepsis may incur poor quality of life and lingering health sequelae such as cognitive impairments, chronic diseases and increased mid-to-long-term mortality for the years following the discharge after the index hospitalization with sepsis (4–7). As a result, the world health organization has recognized sepsis as a global health priority (2). However, effort to reduce sepsis-related morbidity and mortality have mostly concentrated on increasing short-term survival, with little emphasis placed on late morbidity and disease trajectories after hospital discharge. As the number of sepsis survivors increases, assessing the risk of late complications becomes critically important (8–13).

The cardiovascular system is one of the important organ systems commonly affected by sepsis and septic shock (14). Whether sepsis-associated cardiovascular dysfunction leads to the occurrence or worsening of cardiovascular diseases is poorly understood. However, recent evidence has shown cardiovascular events to occur frequently in patients with sepsis during and shortly after admission, with greatest risk occurring during hospital admission (15, 16). In addition, studies have suggested that the increased risk of mortality following sepsis hospitalization cannot be explained solely by poor pre-hospitalization health and might be attributed to increase in-hospital and post-hospitalization incidence of cardiovascular complications, such as arrhythmias, ischemic heart diseases, and stroke (15, 17). Several observational studies have been conducted to investigated the risk of late cerebrovascular events in sepsis survivors. While numerous studies generally suggested an increase in risk of late cerebrovascular events following sepsis, effect estimates have varied extensively. Moreover, the risk factors for adverse cerebrovascular outcomes are not currently well defined. Thus, we sought to conduct a contemporary systematic review and meta-analysis to determine the association between surviving sepsis hospitalization and cerebrovascular complications and to identify and summarize potential risk factors for post-sepsis cerebrovascular complications in adult sepsis survivors.

## Method

### Search strategy and study selection

We conducted the literature search by screening MEDLINE, EMBASE, Web of Science, Scopus, Google Scholar, and Cochrane Controlled library databases using common keywords related to sepsis and cerebrovascular events in September 2022 for studies published from the inception of each database until 31 August 2022. The search was run a second time before finalizing the retrieved articles and incorporating any additional identified studies. Only articles published in English were considered. We included observational studies conducted to evaluate cerebrovascular-related adverse clinical outcomes in adult sepsis survivors compared to the non-septic hospital or population control and studies that identified risk factors for post-sepsis cerebrovascular complication. No restriction was implemented regarding the sex of the patients. According to the selection and exclusion criteria, two researchers independently screened titles and abstracts of search results retrieved from the databases. The full-text articles were obtained for further evaluation for the abstracts identified as potentially relevant by one or both researchers. The same researchers independently reviewed full-text articles for eligibility. All reviewed and excluded articles were documented on an excel spreadsheet with annotations for reasons of exclusion. In duplicate reporting or shared in more than one study, the first published article with the largest sample of patients was included in the analysis. In case of any discrepancies, it was resolved by discussion with a third researcher.

### Data extraction and assessment of quality of the included studies

Two researchers extracted data from the final set of included studies for study characteristics, Baseline population characteristics, and results in a standardized evidence table. The third researcher checked these data for accuracy. Disagreements were managed through discussion between authors. We assessed the methodological quality of the included studies using the Quality in Prognostic Factor Studies (QUIPS) checklist (18). This checklist examines the risk of bias across six domains: study participation, study attrition, prognostic factor measurement, outcome measurement, study confounding, and statistical analysis and reporting. Ratings for each study were compared between the two evaluators, and discrepancies were resolved by consensus.

### Statistical analysis

Observational studies conducted to evaluate cerebrovascular-related adverse clinical outcomes in adult sepsis survivors compared to the non-septic hospital or population control were included in the meta-analysis. For the analysis of the risk of cerebrovascular events following sepsis hospitalization, hazard ratio (HR) with 95% confidence interval (CI) for time-to-event outcomes and odds ratio (OR) for binary outcomes were extracted, from a multivariable model adjusted for confounders. ORs and HRs were combined to summaries the estimates, assuming low incidence of the primary outcome (less than 10%) in the unexposed group (19). When possible, measures of association calculated with hospitalized (as opposed to population) control were prioritized. For the meta-analysis of potential risk factors associated with long-term cerebrovascular complications following sepsis hospitalization, OR from logistic regression and HR from Cox regression were combined because they are closely approximate each other (20–24). For reporting, pooled adjusted effect estimates for each predictor are subsequently referred to as HR with 95% CI. Statistical heterogeneity between studies was evaluated using Chi-squared test (threshold *P* < 0.10) and *I*^2^-statistics, which quantifies the proportion of variance explained by between-study heterogeneity. *I*^2^ ranges between 0.0 and 100.0%. The pooled adjusted estimates were calculated with the Mantel-Haenszel fixed-effect models and the generic inverse variance method. When a significant heterogeneity was identified (*I*^2^ > 50% and *P* < 0.10) (25–27), DerSimonian-Laird random-effects models were used. We used the visual inspection of funnel plots and Egger’s regression test of asymmetry to assess potential publication bias. A series of sensitivity analyses were conducted, including subgroup analyses and meta-regression to investigate the potential source of heterogeneity. Data were stratified according to control for subjects (hospitalized vs. population-based) and effect estimates reported. Subgroup analysis was extended by random-effect meta-regression analysis that allowed the effect of contentious covariates to be investigated [such as in years; mean follow-up time and mean age, and in percent; male gender, hypertension (HTN), debates Mellitus (DM), coronary artery disease (CAD), congestive heart failure (CHF), cerebrovascular accident (CVA), atrial fibrillation (AF), chronic kidney disease (CKD), chronic lung disease (CLD), peripheral vascular disease (PVD), cancer, acquired immunodeficiency syndrome (AIDS), and statin prescription rate]. Additionally, we performed sensitivity analyses to investigate each study’s influence by omitting each in turn from the meta-analysis to test the robustness of the analysis. We followed the recommendation of Meta-analysis of Observational Studies in Epidemiology guideline (MOOSE) (28) and the preferred Reporting Items for Systematic Reviews and Meta-Analyses (PRISMA) (29) 2020 in reporting the present study. We used Review Manager statistical software [RevMan (Computer program) Version 5.4, The Cochrane Collaboration, 2020] and Comprehensive Meta-Analysis statistical software [CMA (Computer program) version 3] (Biostat Inc., Englewood, NJ, USA). A two-sided *P*-value < 0.05 was considered statistically significant.

## Results

### Study selection

**FIGURE 1:**
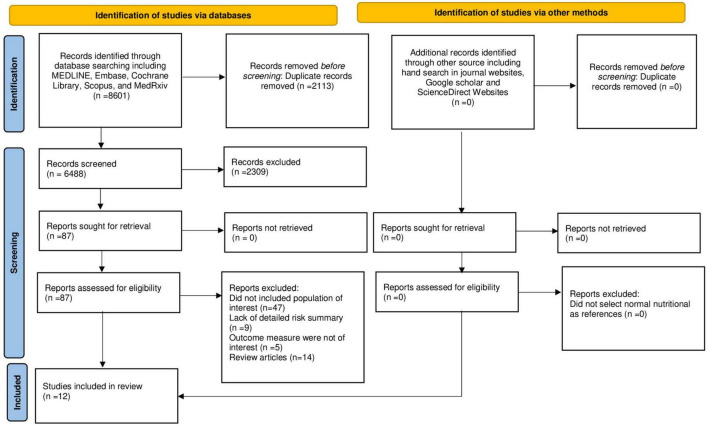
Prisma flowchart showing studies selection process.

The literature search, selection, and reviewing process are depicted in the PRISMA flow diagram (Figure 1). A total of 8,601 references were retrieved during databases and bibliographies search. We excluded 2,113 duplicated publications and an additional 7,345 articles that did not fulfill the selection criteria. After reviewing the full text of the remaining 87 articles, 75 articles were excluded for several reasons, as shown in Figure 1. We included 12 observational studies (17, 30–40) in the final analysis. Among these studies, seven (17, 31–33, 36–38) provided data on the risk of post-sepsis cerebrovascular events and six (30, 33–35, 39, 40) provided data on risk factors associated with the occurrence of cerebrovascular complication following sepsis hospitalization.

### Study characteristics

Relevant study characteristics of the included studies are reported in Table 1. The included studies were published from 2005 to 2019. Eight studies were on Asian population and four were on North American population. The quality assessment of reviewed studies is presented in Supplementary Table 2. The assessment suggested a low-moderate risk of bias for each item. The most common reason for studies having a moderate risk of bias was an inadequate adjustment for confounders and inadequate statistical analyses.

**TABLE 1:** Main characteristics of included studies.

First author, Publication year	Country	Overall sample size	Control subjects	Sepsis definition	Study design	Follow up, years	Age in years	Male, %	HTN, %	DM, %	CAD, %	CHF, %	CVA, %	AF, %	CKD, %	CLD, %	PVD, %	Cancer, %	AIDS, %	Statin, %
Boehm et al. (33)	USA	50194	Population	ICD-9-CM codes	Case-crossover	1	70.4	49	78.3	32	-	13.07	–	–	14	13.7	8.11	–	–	–
Cheng et al. (34)	Taiwan	7419	Hospital	ICD-9-CM codes	Retrospective cohort	4.59	69.79	53.25	36.13	33.71	18.3	9.87	–	2.45	25.12	24.46	–	–	–	–
Cheng et al. (34)	Taiwan	68324	Hospital	ICD-9-CM codes	Retrospective cohort	–	75.06	52.17	32.53	33.1	10.51	11.69	–	–	12.23	16.77	1.86	6.74	–	–
Hsieh et al. (40)	Taiwan	42316	Hospital	ICD-9-CM codes	Prospective cohort	0.5	70.36	59.08	47.75	30.27	2.81	15.82	14.74	1.32	13.43	5.17	3.91	18.67	–	–
Ishani et al. (30)	USA	2311	Population	ICD-9-CM codes	Prospective cohort	3	63	53.29	–	53.81	2.42	44.69	16.77	-	–	–	25.38	–	–	–
Lee et al. (31)	Taiwan	23027	Population	ICD-9-CM codes	Retrospective cohort	10	55.16	53.71	47.4	31.66	28.89	–	–	4.32	–	–	–	–	–	–
Lai et al. (37)	Taiwan	49952	Hospital	ICD-9-CM codes	Retrospective cohort	0.5	66.86	58.06	–	22	5.06	21.06	32.85	–	18.79	51.02	9.89	7.64	0.14	–
Ou et al. (32)	Taiwan	85710	Hospital	ICD-9-CM codes	Retrospective cohort	6.7	56.8	51.31	42	27.2	26.72	9.28	17.58	2.81	12.92	–	–	15.82	0.12	2.27
Sebastian et al. (38)	USA	164209	Population	ICD-9-CM codes	Case-crossover	1	–	–	–	–	–	–	–	–	–	–	–	–	–	–
Shao et al. (39)	USA	121947	Hospital	ICD-9-CM codes	Retrospective cohort	1	65.25	50.03	57.18	36.91	–	20	-	14.8	26.2	23.38	8.9	7.98	0.2	–
Shih et al. (36)	Taiwan	130530	Hospital	ICD-9-CM codes	Retrospective cohort	2.5	70.95	52.64	91.4	67.27	77.32	47.36	56.55	–	32.16	–	–	27.48	-	5.8
Wu et al. (17)	Taiwan	83567	Hospital	ICD-9-CM codes	Retrospective cohort	1	67.84	59.24	–	54	5.92	25.12	37.19	–	19.59	53.93	11.83	25	0.15	–

DM, Diabetes Mellitus; HTN, Hypertension; CAD, Coronary artery disease; CKD, Chronic kidney disease; PVD, Peripheral vascular disease; CHF, Chronic heart failure; CVA, cerebrovascular accident; CLD, Chronic lung disease; AF, Atrial fibrillation; ICD-9-CM codes, International Classification of Diseases Clinical Modification, 9th Revision.

### Post-sepsis risk of cerebrovascular events

A total of seven observational studies (five cohort and two case-crossover) were analyzed to determine the association between sepsis and long-term cerebrovascular events. The quantitative synthesis comprised 587,189 participants. The mean (SD) age of participants was 64.71 (8.04) years, and 51.05 (5.55)% were male. The average follow-up period was 2.90 (3.10) years.

**FIGURE 2:**
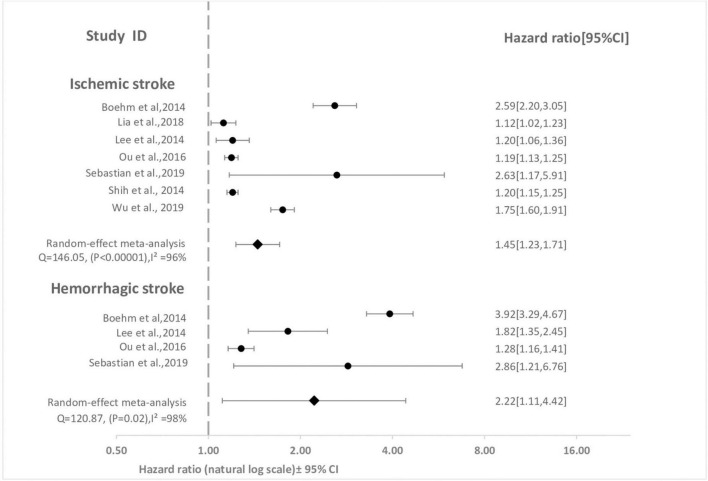
Forest plot for meta-analysis of cohort studies comparing effect of sepsis with no sepsis on stroke. The association between sepsis and each stroke subtype at maximum reported follow-up is pooled and displayed. Weight are from random effect analysis. CI, confidence interval; HR; hazard ratio.

**FIGURE 3:**
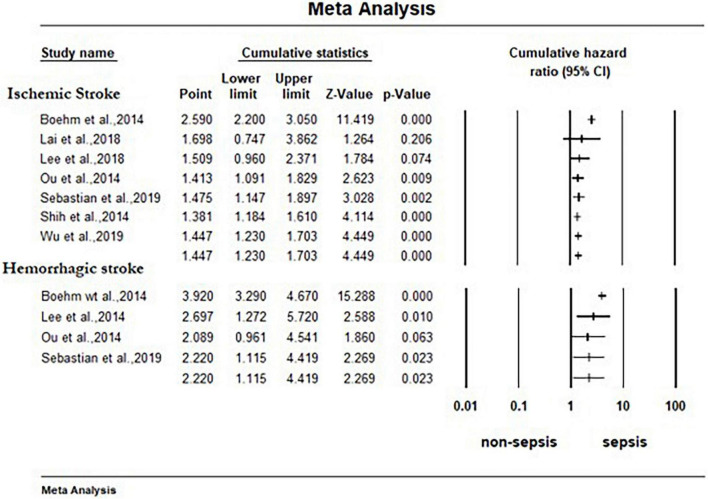
Forest plot for cumulative random effects meta-analysis of cohort studies comparing effect of sepsis with no sepsis on stroke. The association between sepsis and each stroke subtype at maximum reported follow-up is pooled and displayed. The studies are shorted by study size, starting with the largest sized. CI, confidence interval.

As shown in Figure 2, surviving sepsis hospitalization was associated with a significantly higher ischemic stroke [HR 1.45 (95% CI, 1.23–1.71)], and hemorrhagic stroke [HR 2.22 (95% CI, 1.11–4.42)] at maximum follow-up compared to non-sepsis hospital or population control. We identified high-level heterogeneity among studies for both outcomes (ischemic stroke, *I*^2^ = 96% and hemorrhagic stroke, *I*^2^ = 98%). Publication bias was not observed in the studies (i.e., funnel plot was symmetric and Egger test was non-significant) [ischemic stroke (*P* = 0.19) and hemorrhagic stroke (*P* = 0.53)] (Supplementary Figure 1). The cumulative meta-analysis starting with the largest study showed no effect, with increasing effect as the smaller studies were accumulated (Figure 3).

According to the pre-specified subgroups analysis, the stratified pooled meta-analyses demonstrated the stability of significant association of sepsis and primary outcomes (Table 2). The result of the meta-regression is presented in Supplementary Table 4. In the meta-regression analysis, none of the covariant significantly associated with the primary outcomes.

**TABLE 2:** Subgroup analysis performed according to patient and study characteristics considered as potential sources of heterogeneity for outcomes.

Outcome	Subgroups (No. studies)	HR (95% CI)	*I*^2^, %	*P*-value
Ischemic	Hospital control (4)	1.29 (1.11, 1.50)	95	0.0008
stroke	Population control (3)	1.94 (1.02, 3.69)	96	0.04
	HR (4)**^1^**	1.19 (1.15, 1.22)	0	<0.00001
	OR (3)	2.18 (1.54, 3.08)	89	<0.0001
Hemorrhagic	Hospital control (1)	1.28 (1.16, 1.41)	N/A	<0.00001
stroke	Population control (3)	2.74 (1.49, 5.03)	89	0.001
	HR (2)	1.48 (1.05, 2.08)	79	0.02
	OR (2)**^1^**	3.87 (3.26, 4.60)	0	<0.00001

^1^Based on fixed effect meta-analysis. N/A, not applicable; CI, confidence interval; HR, hazard ratio.

Sensitivity analysis showed that the overall effect remained statistically significant when the individual studies were omitted from the effect size calculation for ischemic stroke. However, for hemorrhagic stroke, the effect reached non-significance in two cases when a study by Lee et al. (31) and Sebastian et al. (38) was omitted (Supplementary Figure 2).

### Risk factors of cerebrovascular events

A total of six observational studies that overall included 243,972 sepsis survivors were quantitatively analyzed to identify and summarize the predictors of long-term cerebrovascular complications following sepsis hospitalization. The mean (SD) age of participants was 64.82 (4.72) years, and 52.93 (3.68)% were male. The average follow-up period was 1.60 (1.6) years.

**FIGURE 4:**
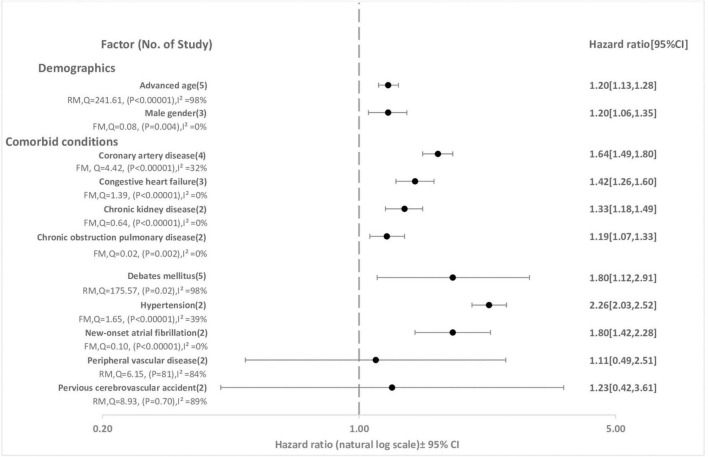
Risk factors for stroke following sepsis hospitalization. FM, fixed effect model; RM, random effect model; CI, confidence interval; HR; hazard ratio.

Figure 4 and Table 3 demonstrate the effect sizes of all 21 factors (un-pooled and pooled) examined as potential risk factors for cerebrovascular complication. A total of 11 factors were present in at least two studies and were included in the pooled meta-analysis. A total of 11 pooled factors that were associated with increased hazards of stroke were: advanced age [HR 1.20 (95% CI, 1.13–1.28)], male gender [HR 1.20 (95% CI, 1.06–1.35)], DM [HR 1.80 (95% CI, 1.12–2.91)], HTN [HR 2.2 (95% CI, 2.03–2.52)], CAD [HR 1.64 (95% CI, 1.49–1.80)], CHF [HR 1.42 (95% CI, 1.26–1.60)], PVD [HR 1.11 (95% CI, 0.49–2.51)], CKD [HR 1.33 (95% CI, 1.18–1.49)], chronic obstruction pulmonary disease (COPD) [HR 1.19 (95% CI, 1.07–1.33)], new-onset atrial fibrillation (NOAF) [HR 1.80 (95% CI, 1.42–2.28)], and pervious cerebrovascular accident (CVA) [HR 1.23 (95% CI, 0.42–3.61)]. Only DM, HTN, CAD, and NOAF were associated with > 50% increase in hazards. Except PVD and pervious CVA, all other factors showed statistically significant effect between the sepsis and control groups. Heterogeneity analysis showed that four pooled risk factors had *I*^2^ greater than 75%. According to egger test, no significant publication was observed for any of the risk factors.

**TABLE 3:** Un-pooled risk factors that were significantly associated with stroke risk following sepsis hospitalization.

Risk factors		ES (95% CI)
**ICU factors**		
	Bed-ridden status	HR 1.51 (1.20–1.90)
**Comorbid condition**		
	Central nervous system dysfunction	HR 1.61 (1.26–2.06)
	Coagulopathy	OR 3.48 (2.93–4.14)
	Hyperglycemic crisis	HR 1.47 (1.18–1.83)
	Hyperlipidemia	OR 2.63 (2.06–3.35)
	Lymphoma	OR 1.60 (1.02–2.51)
	Pulmonary circulation disorders	OR 1.65 (1.22–2.24)
	Valvular heart diseases	OR 1.53 (1.18–1.98)
**Sepsis-related factors**		
	Intra-abdomen infection	HR 1.94 (1.71–2.20)
	Lower respiratory tract infection	HR 1.62 (1.43–1.85)

ES, Effect size; OR, odd ratio; HR, hazard ratio; CI, confidence interval.

Un-pooled factors associated with > 50% increase in risk of stroke included hyperlipidemia [OR 2.63 (2.06–3.35)], bed-ridden status [HR 1.51 (1.20–1.90)], central nervous system dysfunction [HR 1.61 (1.26–2.06)], intra-abdomen infection [HR 1.94 (1.71–2.20)], lower respiratory tract infection [HR 1.62 (1.43–1.85)], coagulopathy [OR 3.48 (2.93–4.14)], lymphoma [OR 1.60 (1.02–2.51)], pulmonary circulation disorders [OR 1.65 (1.22–2.24)], and valvular heart diseases [OR 1.53 (1.18–1.98)] (Table 3).

## Discussion

This systematic review and meta-analysis was conducted to assess whether sepsis-related hospitalization was associated with an increased risk for developing cerebrovascular events and to identify potential risk factors for post-sepsis cerebrovascular complications in adult sepsis survivors. Our analysis showed a significantly increased risk of cerebrovascular events following sepsis hospitalization when compared with the hospital or population control. The increased risk was robust to several sensitivity analyses. Factors that were significantly associated with cerebrovascular complications were: advanced age, male gender, DM, HTN, CAD, CHF, CKD, COPD, and NOAF.

Both acute and chronic infections and inflammatory states have long been implicated in the occurrence and progression of stroke (41–43). In particular, it has been proposed that acute infection, usually respiratory and of bacterial origin may act as a trigger and increase the risk of large vessels and/or cardiometabolic ischemic stroke, especially in patients without vascular risk factors (42, 44). The role of chronic infection in overall stroke risk might be small, however, can increase risk when associated with conventional stroke risk factors, such as HTN, DM, smoking and cardiac diseases, and genetic predisposition (45, 46).

In individuals with sepsis or septic shock, the role of the immune system with subsequent inflammation has been extensively studied (14, 47, 48). Persistent systemic inflammation induced by various infectious agents is associated with vascular disease (41, 49, 50), and elevated circulating concentration of pro-inflammatory biomarkers, catecholamine release, and multi-organ damage during sepsis may contribute to chronic systemic inflammation (48). Inflammation plays a crucial role in the initiation and progression of atherosclerosis and in the development of its acute clinical manifestations (51, 52). Sepsis, with its consequent persistent state of systemic inflammation, may convert stable atherosclerotic plaques to vulnerable plaques, lead to plaque rupture, and increase the risk of subsequent ischemic events (53–56). Similarly, in septic patients activation of the extrinsic coagulation pathway triggered by tissue factors induces increased coagulation activity in the circulation (57, 58). On the other hand, increased production of plasminogen activators inhibitor-1, which suppresses fibrinolytic activity, decreased circulating level of anti-thrombin III and protein C, and reduced thrombomodulin on the endothelial surface, accelerate the pro-coagulation changes (59). In this state, generated intravascular fibrin clots or thrombi may contribute to the formation or progression of atherosclerosis and plaque rupture and may lead to stroke (57, 60, 61). In addition, organ dysfunctions occurred in patients with sepsis or septic shock may persist during recovery and may increase the risk of cerebrovascular disease. For example, septic acute kidney injury may increase the risk of CKD and increase cerebrovascular complications (62–64).

New-onset atrial fibrillation is a well-known complication in critically ill patients, with a reported incidence that varies from 4 to 50% (65–68). According to a systematic review by Kuipers et al. and his colleagues (65), the weighted mean incidence of NOAF was 8%, ranging from 0 to 14% in patients with sepsis, 10%, ranging from 4 to 23% in patients with severe sepsis and 23%, ranging from 6 to 46% in patients with septic shock and was shown to be associated with significant morbidity and mortality. In our study, NOAF during sepsis hospitalization was associated with 1.8-fold increase in hazards for post-sepsis stroke. Similarly, a study by Walkey and his colleagues (16) have presented observational data from large administrative database and have demonstrated that patients with sepsis and NOAF had an increased odd of in-hospital stroke. It is well known that atrial fibrillation is associated with an increased risk of stroke. Nonetheless, the potential mechanisms that might explain the increased risk of stroke in these patients group remain unclear. Although NOAF in the critically ill is often transient, in patients with sepsis, due to cardiac involvement (14, 69), may not resolve and may be a potential source of cardio-embolic stroke. Furthermore, in the presence of other sepsis related factors, such as coagulopathy, NOAF might play a major role in stroke occurrence (39, 70, 71).

Our study has potential implications for clinical practice and future research. Identification of hospitalization with sepsis as a stroke trigger may provide appropriate time to calculate a patient’s cardiovascular risk profile. Identified risk factors among patients with sepsis for a cerebrovascular complication, can help clinicians to consider treating those sepsis patients at height risk with more aggressive treatment with standard preventive strategies. Moreover, our findings should promote clinical trials to test vasculoprotective strategies in this population. Finally, the association of sepsis with cerebrovascular disease risk should also be considered when estimating the cost and benefit of interventions to prevent sepsis.

Our study must be viewed in the context of certain important limitations. First, we incorporated only observational studies; all limitation of observations has to be considered. Second, there was variability in the definition of sepsis severity. In most cases, sepsis was defined using the International Classification of Diseases, Ninth Revision, Clinical Modification (ICD-9-CM), rather than clinical diagnostic, although the accuracy of these codes has been validated. None of the studies included in our study identified sepsis using the current Sepsis-3 guidelines. Further research is needed to corroborate the risk of stroke and identify risk factors using the sepsis definition with the current Sepsis-3 definition. Third, we combined variable control subjects (Hospital-based/population-based), follow-up timeframes, and effect size matrices (HR/OR), due to this, high-level heterogeneity for risk analysis and low to high-level for risk factor analysis was observed among included studies. Thus, the analysis should be interpreted cautiously. Fourth, Information regarding preventive medication and adherence to administered medication during follow-up is not available. Especially, information on the efficacy of antithrombotic therapy, for ischemic stroke prevention in patients with NOAF was not available. Therefore, it remains unclear whether early initiation of prophylactic therapy can prevent cerebrovascular complications. Finally, most studies were susceptible to the competing risk of death, which could contribute to a lower cumulative incidence of cerebrovascular events, particularly in the first year of follow-up (17). Future cohort studies may incorporate proper analytical strategies that take competing risks into consideration (72).

## Conclusion

Our finding showed a significant association between sepsis and subsequent risk of cerebrovascular events. The risk of cerebrovascular events can be predicated by patient and sepsis related baseline variable. New therapeutic strategies are needed for the high-risk patients.

## Data availability statement

The original contributions presented in this study are included in the article/Supplementary material, further inquiries can be directed to the corresponding author.

## Author contributions

AA, BT, GA, AV-F, BA, and DS: study conception, design, and revising the article critically for important intellectual content. AA and BT: acquisition of data, analysis, and interpretation of data. AA: statistical analysis and drafting the article. All authors read and approved the final version of the study to be published.
