# Supplementary material for: Long-term risk and predictors of cerebrovascular events following sepsis hospitalization: A systematic review and meta-analysis
Source: Front Med (Lausanne). 2022 Nov 25;9:1065476. doi: 10.3389/fmed.2022.1065476 (PMC9732021; doi:10.3389/fmed.2022.1065476)
Supplement: Supplementary file 1 [file Data_Sheet_1.pdf]

Supplementary Material

		Pages
**Supplementary Table S1**	Search strategy and inclusion/exclusion criteria	2-10
**Supplementary Table S2**	Quality Assessments of the Included Studies	11
**Supplementary Table S3**	Variables controlled for by propensity score or multivariate analysis	12-13
**Supplementary Figure S1**	Funnel plot of the seven studies included in the meta-analysis of risk of ischemic and hemorrhagic stroke following sepsis hospitalization	14
**Supplementary Table S4**	Meta-regression model between risk of ischemic and hemorrhagic stroke and different patient and study level variables	15-16
**Supplementary Figure S2**	Influential analysis when one study is omitted at the time	17

Table S1: Search strategy and inclusion/exclusion criteria

Database: Ovid MEDLINE(R) and Epub Ahead of Print, In-Process, In-Data-Review & Other Non-Indexed Citations and Daily 1946 to Present

#	Searches
1	Sepsis/
2	Shock, Septic/
3	exp Shock/
4	exp Bacteremia/
5	Capillary Leak Syndrome/
6	Endotoxemia/
7	Hemorrhagic Septicemia/
8	Sepsis-Associated Encephalopathy/
9	exp Systemic Inflammatory Response Syndrome/
10	(Acute adj2 infection*).mp
11	(bacter* adj2 shock).mp.
12	(blood* adj2 poison*).mp.
13	(capillar* adj2 leak*).mp.
14	(endotox* adj2 shock).mp.
15	(h?emorrhag* adj2 shock*).mp.
16	(hemorrhag* adj2 septic*).mp.
17	(infect* adj2 trigger*3).mp
18	(sep*3 adj2 associated adj2 deliri*).mp.
19	(sep*3 adj2 associated adj2 encephalopath*).mp

20	(Septic adj2 shock).mp.
21	(Severe adj2 infect*).mp.
22	(Severe adj2 inflammation*).mp.
23	(Shock adj2 syndrom*).mp.
24	(toxi* adj2 shock).mp.
25	bacteraemi*.mp.
26	bacteremi*.mp.
27	endotoxaemi*.mp.
28	endotoxemi*.mp.
29	pneumonia?.mp.
30	postseps#s.mp.
31	postseptic*.mp.
32	post-seps#s.mp.
33	post-septic*.mp.
34	sepsis.mp.
35	septic.mp.
36	septicaemi*.mp.
37	septicemi*.mp.
38	sirs.ti,ab.
39	systemic inflammatory response syndrome.mp.
40	1 or 2 or 3 or 4 or 5 or 6 or 7 or 8 or 9 or 10 or 11 or 12 or 13 or 14 or 15 or 16 or 17 or 18 or 19 or 20 or 21 or 22 or 23 or 24 or 25 or 26 or 27 or 28 or 29 or 30 or 31 or 32 or 33 or 34 or 35 or 36 or 37 or 38 or 39 40

41	Cardiovascular Diseases/
42	exp stroke/
43	((stroke or strokes) adj8 (acute or cerebr* or cardia* or cardio* or brain? or isch?emi* or vascular)).mp
44	"Hemorrhagic stroke ".mp. or Hemorrhagic stroke /
45	"Ischemic Stroke".mp. or Ischemic Stroke/
46	"Transient Ischemic Attack ".mp. or Transient Ischemic Attack /
47	(cardiovascular* adj1 event?).mp
48	(cardiovascular* adj2 complication?).mp.
49	(Cerebrovascular adj1 accident?).mp.
50	(Cerebrovascular* adj2 complication?).mp.
51	(Cerebrovascular* adj1 event?).mp
52	41 or 42 or 43 or 44 or 45 or 46 or 47 or 48 or 49 or 50 or 51
53	40 and 52
54	limit 53 to English language

Database: Embase 1974 to Present

#	**Searches**
1	Sepsis/
2	Shock, Septic/
3	exp Shock/
4	exp Bacteremia/
5	Capillary Leak Syndrome/
6	Endotoxemia/
7	Hemorrhagic Septicemia/
8	Sepsis-Associated Encephalopathy/
9	exp Systemic Inflammatory Response Syndrome/
10	(Acute adj2 infection*).mp
11	(bacter* adj2 shock).mp.
12	(blood* adj2 poison*).mp.
13	(capillar* adj2 leak*).mp.
14	(endotox* adj2 shock).mp.
15	(h?emorrhag* adj2 shock*).mp.
16	(hemorrhag* adj2 septic*).mp.
17	(infect* adj2 trigger*3).mp
18	(sep*3 adj2 associated adj2 deliri*).mp.
19	(sep*3 adj2 associated adj2 encephalopath*).mp
20	(Septic adj2 shock).mp.

21	(Severe adj2 infect*).mp.
22	(Severe adj2 inflammation*).mp.
23	(Shock adj2 syndrom*).mp.
24	(toxi* adj2 shock).mp.
25	bacteraemi*.mp.
26	bacteremi*.mp.
27	endotoxaemi*.mp.
28	endotoxemi*.mp.
29	pneumonia?.mp.
30	postseps#s.mp.
31	postseptic*.mp.
32	post-seps#s.mp.
33	post-septic*.mp.
34	sepsis.mp.
35	septic.mp.
36	septicaemi*.mp.
37	septicemi*.mp.
38	sirs.ti,ab.
39	systemic inflammatory response syndrome.mp.
40	1 or 2 or 3 or 4 or 5 or 6 or 7 or 8 or 9 or 10 or 11 or 12 or 13 or 14 or 15 or 16 or 17 or 18 or 19 or 20 or 21 or 22 or 23 or 24 or 25 or 26 or 27 or 28 or 29 or 30 or 31 or 32 or 33 or 34 or 35 or 36 or 37 or 38 or 39 40
41	Cardiovascular Diseases/

42	exp stroke/
43	((stroke or strokes) adj8 (acute or cerebr* or cardia* or cardio* or brain? or isch?emi* or vascular)).mp
44	"Hemorrhagic stroke ".mp. or Hemorrhagic stroke /
45	"Ischemic Stroke".mp. or Ischemic Stroke/
46	"Transient Ischemic Attack ".mp. or Transient Ischemic Attack /
47	(cardiovascular* adj1 event?).mp
48	(cardiovascular* adj2 complication?).mp.
49	(Cerebrovascular adj1 accident?).mp.
50	(Cerebrovascular* adj2 complication?).mp.
51	(Cerebrovascular* adj1 event?).mp
52	41 or 42 or 43 or 44 or 45 or 46 or 47 or 48 or 49 or 50 or 51
53	40 and 52
54	limit 53 to English language

Database: Scopus (from inspection to present)

#	**Searches**
1	TITLE-ABS-KEY ("Septic Shock")
2	TITLE-ABS-KEY ("Septicemia")
3	TITLE-ABS-KEY ("Capillary Leak Syndrome")
4	TITLE-ABS-KEY ("Endotoxemia")
5	TITLE-ABS-KEY ("Hemorrhagic Septicemia")
6	TITLE-ABS-KEY ("Pneumonia")
7	TITLE-ABS-KEY ("Sepsis-Associated Encephalopathy")
8	TITLE-ABS-KEY ("sepsis")
9	TITLE-ABS-KEY ("septic")
10	TITLE-ABS-KEY ("systemic inflammatory response syndrome")
11	1 or 2 or 3 or 4 or 5 or 6 or 7 or 8 or 9 or 10
12	TITLE-ABS-KEY ("cardiovascular diseases")
13	TITLE-ABS-KEY ("Stroke")
14	TITLE-ABS-KEY ("Hemorrhagic stroke")
15	TITLE-ABS-KEY ("Ischemic Stroke ")
16	TITLE-ABS-KEY ("Transient Ischemic Attack “)
17	TITLE-ABS-KEY ("cardioembolic stroke")
18	TITLE-ABS-KEY ("cerebrovascular accident")
19	TITLE-ABS-KEY ("Cerebrovascular complication* ")
20	TITLE-ABS-KEY ("Cerebrovascular event*")

21	TITLE-ABS-KEY ("Cardiovascular complication* ")
22	TITLE-ABS-KEY ("Cardiovascular event*")
23	12 or 13 or 14 or 15 or 16 or 17 or 18 or 19 or 20 or 21 or 22 or 23
24	11 and 23
25	limit 24 to English language

Database: Web of sciences (from inspection to present)

#	**Searches**
1	TS="Septic Shock"
2	TS=Septicemia
3	TS="Capillary Leak Syndrome"
4	TS=Endotoxemia
5	TS="Hemorrhagic Septicemia"
6	TS=Pneumonia
7	TS="Sepsis-Associated Encephalopathy"
8	TS=sepsis
9	TS=septic
10	TS="systemic inflammatory response syndrome"
11	1 or 2 or 3 or 4 or 5 or 6 or 7 or 8 or 9 or 10
12	TS="cardiovascular diseases"
13	TS=Stroke
14	TS="Hemorrhagic stroke"

15	TS="Ischemic Stroke"
16	TS="Transient Ischemic Attack"
17	TS="cardioembolic stroke"
18	TS="cerebrovascular accident"
19	TS="Cerebrovascular complication*"
20	TS="Cerebrovascular event*"
21	TS="Cardiovascular complication*"
22	TS="Cardiovascular event*"
23	12 or 13 or 14 or 15 or 16 or 17 or 18 or 19 or 20 or 21 or 22 or 23
24	11 and 23
25	limit 24 to English language

Cochrane Controlled library and Google scholar

(("Septic Shock") OR ("Septicemia") OR ("Capillary Leak Syndrome") OR ("Endotoxemia") OR ("Hemorrhagic Septicemia") OR ("Pneumonia") OR ("Sepsis-Associated Encephalopathy") OR ("sepsis") OR ("septic") OR ("systemic inflammatory response syndrome")) AND (("cardiovascular diseases") OR ("Stroke") OR ("Hemorrhagic stroke") OR ("Ischemic Stroke ") OR ("Transient Ischemic Attack “) OR ("cardioembolic stroke") OR ("cerebrovascular accident") OR ("Cerebrovascular complication* ") OR ("Cerebrovascular event*") OR ("Cardiovascular complication* ") OR ("Cardiovascular event*"))

Table S2: Quality assessment according to Quality in prognostic Factor Studies (QUIPS) checklist for individual studies.

Study	Publication year						
		Study participation	Study attrition	Prognostic factor measurement	Outcome measurement	Study confounding	Statistical analysis and reporting
Boehm	2014	low	low	low	low	moderate	low
Cheng	2017	low	low	low	low	moderate	moderate
Cheng	2017	low	low	low	low	moderate	moderate
Hsieh	2019	low	low	low	low	low	moderate
Ishani	2005	low	low	moderate	low	moderate	moderate
Lai	2018	low	low	low	low	moderate	low
Lee	2014	low	low	low	low	moderate	moderate
Ou	2016	low	low	low	low	moderate	low
Sebastian	2019	low	low	low	low	moderate	low
Shao	2019	low	low	low	low	moderate	low
Shih	2014	low	moderate	low	low	moderate	moderate
Wu	2019	low	low	low	low	moderate	low

Note: A study can be awarded a maximum of one star for each numbered items within the selection and outcomes categories. A maximum of two stars can be given for comparability

Table S3. Variables controlled for by propensity score or multivariate analysis

First author	Pub year	Control for confounding	Age	Sex	HTN	DM	CAD	CHF	CVA	AF	CKD	CLD	PVD	Cancer	AIDS	Statin	Additional medication
**Boehm**	2014	Multivariate	*	*	*	*					*	*	*	*			
**Cheng**	2017	Multivariate	*	*	*	*	*	*			*	*	*	*			
**Cheng**	2017	Multivariate	*	*	*	*	*	*		*	*	*					
**Hsieh**	2019	Multivariate	*	*	*	*	*	*	*	*	*	*	*	*	*		
**Ishani**	**2005**	Multivariate	*	*		*	*	*	*	*	*		*				
**Lia**	**2018**	PS matched	*	*		*	*	*	*		*	*	*	*	*		
**Lee**	**2014**	Multivariate	*	*	*	*	*			*							
**Ou**	**2016**	PS matched	*	*	*	*	*	*	*	*	*			*	*	*	*
**Shao**	2019	Multivariate	*	*	*	*		*		*	*	*	*	*	*		
**Shih**	**2014**	PS matched	*	*	*	*	*	*	*		*			*		*	*
**Wu**	**2019**	PS matched	*	*		*	*	*	*		*	*	*	*	*		

First author	Additional variables
**Boehm**	Number of chronic conditions, length of stay(days), race, valvular disease, coagulopathy, psychoses, paralysis, other neurological disorders, rheumatoid arthritis/collagen vascular diseases and alcohol abuse
**Cheng**	Type of organ failure (circulatory, hematologic, hepatic, metabolic, neurologic, and renal Respiratory), number of organ failure, CCI, and type of infection (Pneumonia, Urinary tract infection, Skin infection, and GI infection)
**Cheng**	Hyperlipidemia and hospital type and area
**Hsieh**	Urbanization level, Insurance premium level, dementia, rheumatologic disease, peptic ulcer disease, chronic liver disease, hemiplegia or paraplegia status, hypercholesterolemia, source of infection (upper respiratory tract infection, lower respiratory tract infection, urinary tract infection, Intra-abdomen infection, and skin and soft tissue infection),and organ dysfunction (central nervous system dysfunction, acute renal failure, septic shock, acute liver failure, acute respiratory failure, and hyperglycemic crisis)
**Ishani**	Dialysis access, primary renal disease, race, serum albumin, and adjusted mortality hazards ratio
**Lia**	Dementia, rheumatologic disease, peptic ulcer disease, liver disease, and hemiplegia or paraplegia status
**Lee**	**-**
**Ou**	Monthly income, urbanization level, CCI, valvular heart disease, and drug abuse
**Sebastian**	-
**Shao**	Valvular heart disease, coagulopathy, rheumatoid arthritis/collagen vascular diseases, obesity, alcohol abuse, drug abuse, depression, and psychoses
**Shih**	Monthly income, urbanization level, CCI, valvular heart disease, ESRD, dyslipidemia, valvular heart disease, and drug abuse
**Wu**	Urbanization level, Insurance premium level, dementia, rheumatologic disease, peptic ulcer disease, liver disease, hemiplegia or paraplegia status, alcohol/drug use, psychiatric disorder, neurologic disorder, obesity, bed-ridden status, solid organ transplantation such as renal or heart transplantation, and healthcare service utilization (number of OPD visits, number of emergency department visits, number of hospital admissions)

Abbreviations: DM: Diabetes Mellitus, HTN: Hypertension, CAD: Coronary artery disease, CKD: Chronic kidney disease, PVD: Peripheral vascular disease, CHF: Chronic heart failure, CVA: cerebrovascular accident, CLD: Chronic lung disease, AF: Atrial fibrillation, GI: Gastrointestinal, SIRS: Systemic inflammatory response syndrome, ICU: intensive care unit, ESRD: End-Stage Renal Disease, OPD: outpatient department

Figure S1: Funnel plot of the seven studies included in the meta-analysis of risk of ischemic and hemorrhagic stroke following sepsis hospitalization

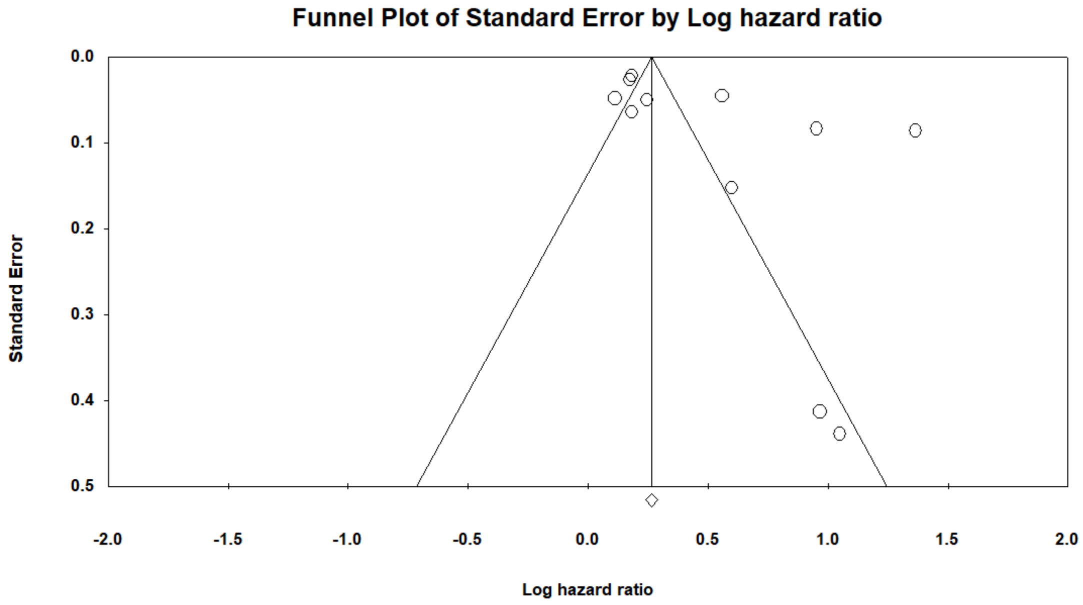

Egger test

Ischemic stroke: Two-sided p-value: 0.19

Hemorrhagic stroke: Two-sided p-value: 0.539

Table S4. Meta-regression model between risk of ischemic and hemorrhagic stroke and different patient and study level variables

Covariant	Outcomes	Coefficient	Lower limit	Upper limit	2-sided P- value[1]
**Mean age**	IS	0.0222	-0.0116	0.0560	0.1985
	HS	-	-	-	
**Male gender**	IS	-0.0225	-0.0178	0.0269	0.3727
	HS	-	-	-	-
**Mean follow up period**	IS	-0.0403	-0.0916	0.0110	0.1232
	HS	-	-	-	-
**Hypertension**	IS	0.0060	-0.113	0.0232	0.4969
	HS	-			
**Debates mellitus**	IS	0.0011	-0.0125	0.0146	0.8781
	HS	-	-	-	-
**Coronary artery disease**	IS	-0.0020	-0.0080	0.0.0040	0.5095
	HS	-	-	-	-
**Congestive heart failure**	IS	-0.0073	-0.0279	0.0133	0.4865
	HS	-	-	-	-
**Cerebrovascular accident**	IS	-	-	-	
	HS	-	-	-	-
**Atrial fibrillation**	IS	-	-	-	-
	HS	-	-	-	-
**Chronic kidney disease**	IS	-	-	-	-
	HS				
**Chronic lung disease**	IS	-	-	-	-
	HS	-	-	-	-
**Peripheral vascular disease**	IS	-	-	-	-

	HS				
**Cancer**	IS	0.0091	-0.0078	0.0259	0.2920
	HS	-	-	-	-
**AIDS**	IS	-	-	-	-
	HS	-	-	-	-
**Statin**	IS	-	-	-	-
	HS	-	-	-	-

[1] Based on random effect model. IS, ischemic stroke; HS, hemorrhagic stroke

Figure S2: Estimates when omitting one study at the time for MI and stroke risk meta-analysis.

Ischemic stroke

Meta Analysis

Study name	Hazard ratio	Lower limit	Upper limit	Z-Value	p-Value
	Statistics for each study				
Boehm et al.,2014	2.590	2.200	3.050	11.419	0.000
Lai et al.,2018	1.120	1.020	1.230	2.373	0.018
Lee et al.,2018	1.200	1.059	1.359	2.868	0.004
Ou et al.,2014	1.190	1.131	1.252	6.756	0.000
Sebastian et al.,2019	2.630	1.170	5.911	2.340	0.019
Shih et al.,2014	1.200	1.151	1.251	8.571	0.000
Wu et al.,2019	1.750	1.602	1.912	12.387	0.000
	1.447	1.230	1.703	4.449	0.000

Hazard ratio and 95% CI

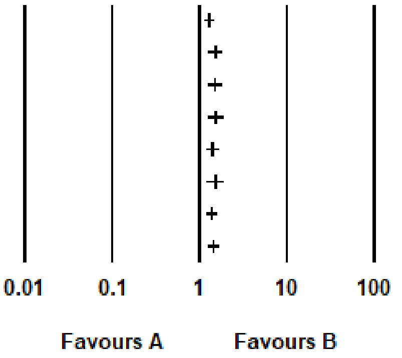

Meta Analysis

Hemorrhagic stroke

Meta Analysis

Study name	Point	Lower limit	Upper limit	Z-Value	p-Value
	Statistics with study removed				
Boehm wt al.,2014	1.625	1.131	2.335	2.628	0.009
Lee et al.,2014	2.392	0.958	5.974	1.867	0.062
Ou et al.,2014	2.737	1.488	5.035	3.238	0.001
Sebastian et al.,2019	2.089	0.961	4.541	1.860	0.063
	2.220	1.115	4.419	2.269	0.023

Hazard ratio (95% CI) with study removed

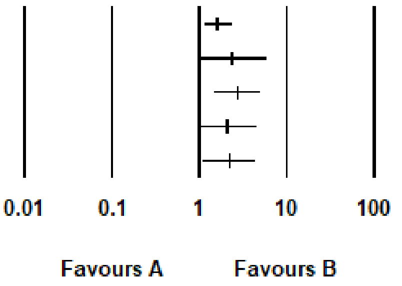

Meta Analysis

Note: CI, confidence interval; HR; hazard ratio
